# Plantamajoside, a potential anti-tumor herbal medicine inhibits breast cancer growth and pulmonary metastasis by decreasing the activity of matrix metalloproteinase-9 and -2

**DOI:** 10.1186/s12885-015-1960-z

**Published:** 2015-12-16

**Authors:** Shimin Pei, Xu Yang, Huanan Wang, Hong Zhang, Bin Zhou, Di Zhang, Degui Lin

**Affiliations:** The Clinical Department, College of Veterinary Medicine, China Agricultural University, Beijing, 100193 China; Department of Veterinary Medicine, College of Animal Sciences, Zhejiang University, Hangzhou, 310058 China

**Keywords:** PMS, Herbal medicine, Breast cancer, Metastasis, MMP9 and MMP2, Angiogenesis

## Abstract

**Background:**

Metastasis is the major cause of death in breast cancers. MMPs play a key role in tumor microenvironment that facilitates metastasis. The existing researches suggest that the high expression of gelatinase A and B (MMP2 and MMP9) promote the metastasis of breast cancer. Therefore, gelatinase inhibitor can effectively suppress tumor metastasis. However, at present, there is no dramatically effective gelatinase inhibitor against breast cancer.

**Methods:**

We screened gelatinase inhibitor among Chinese herbal medicine by molecular docking technology; investigated the proliferation, migration and invasion of MDA-MB-231 human breast cancer cell line and 4T1 mouse breast cancer cell line in response to the treatment with the screened inhibitor by wound assay, invasion assay and gelatin zymography; then further examined the effects of inhibitor on allograft mammary tumors of mice by immunohistochemistry.

**Results:**

We successfully screened an Chinese herbal medicine-Plantamajoside(PMS)-which can reduce the gelatinase activity of MMP9 and MMP2. In vitro, PMS can inhibit the proliferation, migration and invasion of MDA-MB-231 human breast cancer cell line and 4T1 mouse breast cancer cell line by decreasing MMP9 and MMP2 activity. In vivo, oral administration of PMS to the mice bearing 4T1 cells induced tumors resulted in significant reduction in allograft tumor volume and weights, significant decrease in microvascular density and significant lower lung metastasis rate.

**Conclusions:**

Our results indicate that as a promising anti-cancer agent, PMS may inhibit growth and metastasis of breast cancer by inhibiting the activity of MMP9 and MMP2.

**Electronic supplementary material:**

The online version of this article (doi:10.1186/s12885-015-1960-z) contains supplementary material, which is available to authorized users.

## Background

Breast cancer is the most common type of malignant disease in women worldwide. In the past two decades the mortality rate in breast cancer patients has been decreasing thanks to the development of early diagnostic methods and more effective treatments. However, breast cancer is still the second leading cause of cancer-related deaths in women [1], the main reason for that is the metastasis. As Christopher R.bohl pointed out, the most deadly attribute of breast cancer cells is their ability to leave their initial site of growth, travel to discontinuous secondary sites, and proliferate into macroscopic masses [2]. What’s more, conventional treatments (e.g. chemotherapy, radiotherapy and surgery) can cure primary tumors, but cannot control the secondary tumors. Many breast cancer patients suffer relapse and metastasis after treatment [3]. The metastasis of primary tumors depends not only on the characteristics of cancer cells themselves, but also the formation of proper environments, which is named as “metastatic niche”. New metastases couldn’t generate before the “pre-metastatic niche” developing into “metastatic niche” [4]. Therefore, in order to cure breast cancer, the mechanism of its metastasis must be fully understood to facilitate the establishment of methods to suppress the metastasis.

Matrix metalloproteinase 9 (MMP9) and Matrix metalloproteinase 2(MMP2) show a stronger expression in breast cancer tissue compared to that in normal breast tissue [5]. MMP9 expresses activity to degrade the extra cellular matrix (ECM) in the vicinity of tumor, which has close relationship with the invasion and metastasis of tumors. The previous research has proved that MMP9 plays a key role in the process of formation of “metastatic niche” and regulates pulmonary metastasis via over expression [6]. MMP9 is treated as a “general commander” of ECM remodeling, in charge of the final degrading of collagenous fiber, releasing tumor cells from the surrounding complicated network. Take mammary gland tissue for instance, collagen composed the major part of connective tissue, its degradation includes two steps—collagenases disintegrate the whole fiber into tiny fragment which can denatured into gelatin, then MMP9 decomposed these gelatins [7].

Mounting researches have shown that MMP9 in serum and tissue can serve as a prognosis biomarker for tumors [8, 9]. The significant overexpression of MMP9 in serum associates with lymph nodes metastasis, higher staging, shorter disease-free time and overall survival time [8, 9]. Human breast cancer cell-produced MMP9 is specifically required for invasion in cell culture and for pulmonary metastasis in a mouse orthotopic model of basal-like breast cancer. MMP9 may offer a target for anti-metastatic therapies for basal-like triple negative breast cancers [10]. MMP-2, MMP-9 and eukaryotic transcription factor-1(ETS-1) co-expression might be used as a poor prognostic factor in breast cancer patients [11]. Stromal MMP-2 expression may play a crucial role in predicting aggressive clinical behavior in breast cancer patients [12]. MMP-2 in stromal fibroblasts might indicate poor survivors in patients with high grade breast cancer [13]. MMPs are not only a prognosis factor for breast cancer, but also a potential treatment target. There are lots of surveys on regulation of MMPs. In the past few decades, the research about MMP inhibitors (MMPIs) in tumor treatment has developed rapidly. The third generation MMPIs is under the clinical research currently. The third MMPIs mainly target on some special MMPs, like MMP9 and MMP2. The original concept that MMPs are just ECM remodeling regulator has been taken place by new concepts that MMPs are proteinase which can regulate the functions of various protein [14]. Furthermore, the substitutes and products of MMPs can also been seen as the target of cancer treatment. All of these researches on cancer treatments must depend on the profound understanding of the role of MMPs in tumor microenvironment, tumor growth and metastasis.

Since MMP9 and MMP2 regulate tumor microenvironment and tumor metastasis, in theory, the invasion and metastasis of tumor can be inhibited through decreasing the activity of MMP9 and MMP2. Nowadays, more and more traditional Chinese medicines (TCMs) are applied in the prevention and treatment of many different kinds of tumors. We have particular advantages of accessing to TCMs, because medicine resource in China is abundant. There are more than 12,800 medical animals and plants TCMs, which provide a wide choice for new anti-tumor drug screening [15]. Docking calculations has been applying in medical research for more than 20 years. Computational approaches that ‘dock’ small molecules into the structures of macromolecular targets and ‘score’ their potential complementarity to binding sites are widely used in hit identification and lead optimization [16]. Plantamajoside(PMS) was selected by combining MMP9 through Docking calculation.

PMS is an extract from Herba Plantaginis, a conventional TCM, with the role of antiviral, diuretic, antioxidant and immune enhancement [17]. It’s been used in medicine and food in a long term. Herba Plantaginis contains polyphenols, mainly phenylpropanoid glycosides and flavonoids. PMS is a unique component for identification of Herba Plantaginis, belongs to phenylpropanoid glycoside. A study showed that the PMS concentration of the peak blood plasma of rat after oral administration was 172.3 ± 35.1 ng/mL, the required time was 16.7 ± 2.8 min [18]. Other studies have indicated that PMS has the antioxidant effect and has the protective effect on the kidney damage caused by cadmium [19]. Despite this, there are no reports of anti-tumor studies on PMS. Since PMS can target combine with MMP9 and MMP2, we assumed that PMS could suppress the growth and metastasis of breast cancer via regulating the activity of MMP9 and MMP2.

## Methods

### Chemical

**Fig. 1:**
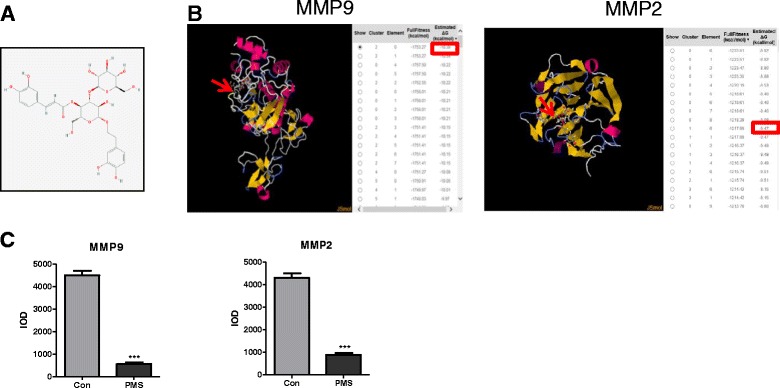
Plantamajoside(PMS) affects the activity of MMP9 and MMP2. **a** A diagram of the structure of PMS(PubChem CID:5281788). **b** The molecular docking of PMS to MMP9 and MMP2. We can see the PMS molecule (indicated with red arrow) combined with MMP9 or MMP2, and the minimum ∆G (circled in red square)value were -10.39 and -9.51 kcal/mol respectively. **c** Detected IOD of substrate activated by MMP9 and MMP2 treated with solvent or PMS. Data represent the mean ± S.D. of three independent experiments. The *** indicates extremely significance different

Plantamajoside (PMS) and other ten agents (Catechin, Chrysophanol, Phlorizin, Salidroside, Curcumin, Isoacteoside, Silymarin, Echinacoside, Gastrodin, Harpagoside) were purchased from CHENGDU MUST BIO-TECHNOL CO., LTD(Sichuan, China), ≥98 %, dissolved in solution with equal proportion of ethanol and ultrapure water when administrated. The chemical structure of Plantamajoside is shown in Fig. 1a.

### MMP activity inhibition in vitro

The affinity of all 11 agents with MMP9 and the affinity of PMS with MMP2 were evaluated through molecular docking technology according to Docking calculations by SwissDock software (Swiss Institute of Bioinformatics, Lausanne, Switzerland). The lower binding free energy(ΔG) is, the stronger combining ability is. Enzyme inhibition test in vitro was performed to detect the inhibition effect of 11 agents on MMP activity. Load 50 μL of 0.4 ng/μL activated Recombinant Human MMP­9 (rhMMP­9) (Catalog # 911­MP,R&D system,Inc. U.S.A) or MMP2(Catalog # 902­MP,R&D system,Inc. U.S.A) and 50 μL of 20 μM Substrate (MCA­Pro­Leu­Gly­Leu­DPA­Ala­Arg­NH) (Catalog#ES001,Catalog # 911­MP,R&D system, Inc.U.S.A) into Black Maxisorp Plate(Nunc, Catalog # 475515, Dermar-k) with solvent (Control) or with 100 μg/mL of each agent to start reaction. Triplicated wells were used for each group. Read the absorbance [present as optical density (OD)] at excitation and emission wavelengths of 320 and 405 nm on Fluorescent Plate Reader (MD spectra-max m5, Molecular Devices, U.S.A). The activity of MMP9 was direct proportional to OD.

### Cell culture

MDA-MB-231(ATCC® HTB-26™) human breast tumor cell line and 4T1 (ATCC® CRL-2539™) mouse breast tumor cell line were purchased from ATCC (American Type Culture Collection, Manassas, VA, USA). Chinese Hamster Ovary (CHO-K1)(Cell bank of Chinese Academy of Science, Beijing, China) . MDA-MB-231 cells and 4T1 cells were grown in DMEM(gibco, life technologies, China) medium or RPMI-1640 (gibco, life technologies, China)medium respectively supplemented with 10 % fetal bovine serum (FBS, gibco, life technologies, China),and penicillin(100units/mL) and streptomycin(100units/mL) incubated at 37 °C in a 5 % CO_2_ –95 % air environment. CHO cells were cultured in the same condition as MDA-MB-231 cells.

### Cell growth evaluation

Cell viability assay was performed by planting cells (both MDA-MB-231 cells and 4T1 cells) in 96-well microplate at a density of 1 × 10^4^ cells/well for 24 h before attached. Then cells were divided in different groups including control group (solvent without PMS) and groups treated with various doses of PMS or Catechin. Triplicated wells were used for each group. Cell viability was assessed with Cell Counting Kit (CCK-8, Beyotime, Shanghai, China.) at 0, 12, 24, 36, 48 h post-treatment according to the manufacturer’s instructions. To determine the cell viability, OD_450_ (the absorbance value at 450 nm) was read with 96-well plate reader (ELx808 Absorbance Reader, BioTek. China). Cell viability assay was also performed on CHO cells to investigate the side effect of PMS on normal cells. The PMS concentration closed to IC_50_ (data not shown) for MDA-MB-231 cells and 4T1 cells at 36 h time point was chosen to treat CHO.

For the colony formation assay, properly resuspend cells were randomly plated in 6-well plate at a density of 1 × 10^4^ cells/well with solvent without PMS (Control) or with 100 μg/mL PMS or Catechin. Triplicated wells were used for each group. After 36 h treatment, washed out the cell debris and nonattached cells, added fresh medium without PMS into all of the wells, followed by 10-day incubation. The attached cells were stained with 0.1 % (W/V) crystal violet (Solarbio, Beijing China).

### Migration assay

Wound assay was performed to evaluate the migration ability of cells. Cells were seeded in 6-well plate and grew to confluence followed by scratching the monolayer cells with a 200 μL pipette tip to create wound. Plates were washed to remove floating cells and debris and then incubated with medium with solvent without PMS (Control) or with 200 μg/mL PMS or Catechin. Triplicated wells were used for each group. Photographed the cells migration images at 0, 36 h. Open wound area (percentage of an image that is not covered by cells) was calculated with the TScratch software(Computational Science & Engineering Laboratory, Zurich, Switzerland).

### Invasion assay

8 μm pore-size tanswell filters(Costar, Corning Incorporated, U.S.A) were put in 24-well plate and the upper chambers were covered with BD Matrigel Matrix(BD,U.S.A), then cells were seeded onto the filters at a concentration of 1 × 10^4^ cells/well in 100 μL of FBS free medium with solvent without PMS (Control) or with different concentration of PMS or Catechin. The lower chambers were filled with 600 μL of medium with 10 % FBS. Triplicated wells were used for each group. After 36 h of treatment, cells on the topside of the filter were removed by scrubbing with a tipped swab. The migration of cells to the lower side of the filter was determined by crystal violet staining. To investigate the relevant of the inhibition effects of PMS in MMPs activity and tumor invasion, exogenous MMP9 (Catalog # 911­MP,R&D system,Inc. U.S.A) contained in fresh media was added to the wells treated 36 h with PMS. After 36 h treatment of exogenous MMP9 at the concentration of 200 ng/mL, the consequent tests described above were performed.

### Gelatin zymography

Cells were randomly plated in 6-well plate at a density of 2 × 10^5^ cells/well with solvent without PMS (Control) or with 125 and 250 μg/mL PMS or with 100 and 200 μg/mL Catechin. Triplicated wells were used for each group. After 24 h or 36 h treatment, washed the cell monolayer with sterile Phosphate Buffered Saline (PBS) to remove the serum completely. Then incubated the cells in serum-free media at 37 °C in a Carbon dioxide (CO_2_)incubator for 12 h. The culture media were collected and centrifuged at 14,000 rpm for 10 min at 4 °C,the protein concentration was determined. Equivalent samples were subjected to sodium dodecyl sulfate-polyacrylamide gelelectrophoresis (SDS-PAGE) on 10 % gel which contained 0.1 % w/v gelatin (Sigma,U.S.A). The gel was removed to renaturing solution [2.5 % Triton X-100, 50 mmol/L Tris-HCl, 5 mmol/L CaCl_2_ and 1 μmol/L ZnCl_2_ in distilled water (dH_2_O)] for 1 h at room temperature with gentle agitation and then was rinsed with dH_2_O completely. Next the gel was incubated in developing solution (50 mmol/L Tris-HCl, 5 mmol/L CaCl_2_, 1 μmol/L ZnCl_2_ and 0.02 % Brij-35 in dH_2_O) for 20 h and stained 3 h in staining solution (0.05 % Coomassie blue RR-250, 30 % methanol and 10 % acetic acid in dH_2_O), followed by destained in destaining solution(5 % methanol and 10 % acetic acid in dH_2_O) until area of gelatinolytic activity appeared as clear sharp bands over the blue background.

### Western blotting

Cells were randomly plated in 6-well plate at a density of 2 × 10^5^ cells/well with solvent or with 125 and 250 μg/mL PMS. Triplicated wells were used for each group. After 36 h treatment, cells were harvested and washed twice with ice-cold phosphate-buffered saline (PBS, PH 7.4), and lysed with ice-cold lysis buffer (P0013B, Beyotime, China) for 30 min on ice. The lysates were centrifuged at 12,000 rpm for 5 min at 4 °C, and the protein concentration was determined. Equivalent samples (20 μg protein extract was loaded on each lane) were subjected to SDS-PAGE on 10 % gel. The proteins were then transferred onto polyvinylidene fluoride (PVDF) membranes (IPVH000 10, MercKMillipore), and probed with indicated primary antibody, MMP9(ab38898, Abcam,1:500), MMP2(sc-13595, Santa Cruz, 1:500) and GADPH (as loading control, sc-166574, Santa Cruz, 1:500). Primary antibody was detected by binding horseradish peroxidase (HRP)-conjugated anti-rabbit or anti-mouse secondary antibody with an Electro-Chemi-Luminescence (ECL) plus kit (32109, Thermo, China).

### Allograft experiment

The animal study was approved by the Institutional Animal Care and Use Committee of China Agricultural University. Subcutaneous inoculation of 1.5 × 10^6^ 4T1 cells in 200 μL PBS was carried out in 4-weeks-old BALB/c mice. The fifth day after inoculation, mice were treated daily with solvent without PMS (Control) (*n* = 6) or with PMS (*n* = 6) at 200 mg/kg body weight by oral delivery. After 21-day treatment, all mice were euthanized for collection of allograft tumors and lungs.

### Immunohistochemical analysis

4T1 allograft tumors and lung tissues were dissected and fixed in 10 % (v/v) neutral-buffer formalin for 24 h. The fixed tissues were dehydrated in ascending grades of ethanol and xylene, and then embedded in paraffin wax. Sections (3 μm) were cut with microtome (Leica, Germany) and mounted on CITOGLAS ® adhesion microscope slides (CITOTEST, Jiangsu, China). Immunostaining was performed by using antibodies for the proliferation marker protein -antigen identified by monoclonal antibody Ki-67 (Ki67) (ZSGB-BIO, Beijing, China), cluster of differentiation 31(CD31) (Bioss, Beijing, China 1:150). The biotinylated secondary antibody was goat anti-rat and anti-rabbit antibody IgG (ZSGB-BIO, Beijing, China). The slides were firstly stained with diaminobenzidine (DAB) and then counter stained with hematoxylin. The stained slides were dehydrated and mounted coverslips with neutral glue. Images were captured and analyzed by Image-pro-plus software (Media Cybernetics, Washington, USA).

### Statistical analysis

Numerical results are expressed as mean ± standard deviation. Treatment effects were compared by analysis of variance or Student’s t-test (when only 2 groups) and differences between means were considered to be significant when *P* <0.05. The analyses were performed using SPSS 20 software (Statistical Product and Service Solutions, Chicago, USA).

## Results

### PMS dramatically reduced the activity of MMP9 and MMP2 in vitro

According to Docking calculations by SwissDock software, PMS can targeted combined to MMP9 (Fig. 1b), the affinity is strongest when ∆G = -10.39 kcal/mol. Other agent can also combine to MMP9 (Additional file 1: Table S1 and Additional file 2: Figure S1), however the ∆G was higher than -10 kcal/mol, which means the affinity of PMS to MMP9 was the strongest among these 11 agents. PMS can also targeted combined to MMP2, but the ∆G was higher than -10 kcal/mol too. In order to detect the inhibition function of these agents towards MMP, enzyme inhibition test in vitro was performed and the result (Fig. 1c) shows that the integrated optical density (IOD) of 100 μg/mL PMS treatment (557.0 ± 67.6 or 890.1 ± 82.2) was significantly lower than that of control group (4499.8 ± 185.9 or 4301.3 ± 211.3)(*P* <0.01) in MMP9 or MMP2 inhibition assay respectively. Therefore, PMS can decrease the activity of MMP9 and MMP2 by a large mount. The inhibition effect of other agents (Additional file 2: Figure S1) are worse than that of PMS.

### Cell proliferation decreased significantly after treatment of PMS

**Fig. 2:**
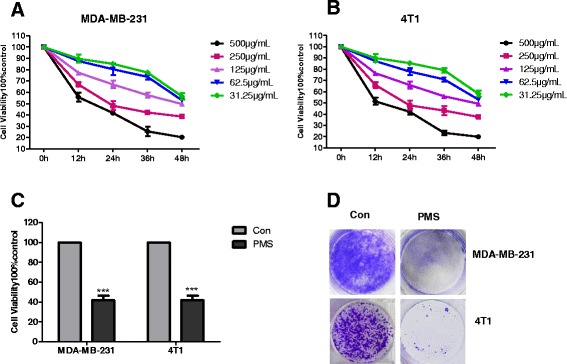
Cell viability decreased after treatment of PMS. Cell viability was analyzed using Cell Counting Kit 8 at 0, 12,24,36,48 h after 31.25, 62.5, 125, 250, 500 μg/m L PMS treatment in (**a**) MDA-MB-231 and (**b**) 4T1 cells. **c** Cell viability was detected after 36 h 250 μg/mL PMS treatment. Data represent the mean ± S.D. of three independent experiments. **d** Colony formation of MDA-MB-231 and 4T1 cells. Cells were treated with 100 μg/mL PMS for 36 h, followed with crystal violet staining of attached cells at 10 days. Similar results were obtained from independent experiments. The *** indicates extremely significance different

To establish a proper treatment dose of PMS, MDA-MB-231 cells and 4T1 cells were treated with various doses of PMS. Cell viability was analyzed using Cell Counting Kit-8 after 0, 12, 24, 36 and 48 h treatment. Cells showed a decrease in viability with increasing dose, and a correlation was observed with post treatment period (Fig. 2a and b), decrease of cell viability treated with PMS is dose and time dependent. PMS shows clear dose and time dependency in both of these two cell lines. As shown in Fig. 2c, cell viability has significant decrease after the 250 μg/mL PMS 36 h treatment (*P* <0.01). CHO cells were treated with 300 μg/ml PMS for 36 h, and there was no significant change of cell viability between pre and post PMS treatment (Additional file 3: Figure S2d), which indicates that PMS has no side effect on normal cells.

Next, the ability of cell colony was evaluated by colony formation assay. MDA-MB-231 cells and 4T1 cells were treated with 100 μg/mL PMS. As shown in Fig. 2d, the crystal violet staining results suggested that PMS remarkably inhibited colony formation of MDA-MB-231 cells and 4T1 cells.

### Cell proliferation decreased significantly after treatment of Catechin

Catechin is one of the ten agents we screened from. It’s also an extract from traditional Chinese medicine as well as PMS. Docking calculation result shows that Catechin also has affinity to MMP9 (Additional file 1: Table S1), but in enzyme inhibition test in vitro, Catechin doesn’t show inhibition effect on MMP9 activity (Additional file 2: Figure S1). So we choose Catechin as the negative control to PMS in the consequent cell experiments.

MDA-MB-231 cells and 4T1 cells were treated with various doses of Catechin. Cell viability was analyzed using Cell Counting Kit-8 after 0, 12, 24, 36 and 48 h treatment. Cells showed a decrease in viability with increasing dose, and a correlation was observed with post treatment period (Additional file 2: Figure S2 A and B), decrease of cell viability treated with Catechin is dose and time dependent. The ability of cell colony was evaluated by colony formation assay. MDA-MB-231 cells and 4T1 cells were treated with 100 μg/mL Catechin. As shown in Additional file 3: Figure S2C, the crystal violet staining results suggested that Catechin remarkably inhibited colony formation of MDA-MB-231 cells and 4T1 cells.

### PMS inhibited cells migration and invasion through down regulated the activity instead of the expression of MMP9 more than that of MMP2

Since migration of tumor cells across the blood vessel-lining endothelial monolayers and their invasion through extra cellular matrix (ECM) play an important role in the metastatic process, effect of PMS was evaluated on the migration and invasive behavior of both human and rodent breast cancer cells.

**Fig. 3:**
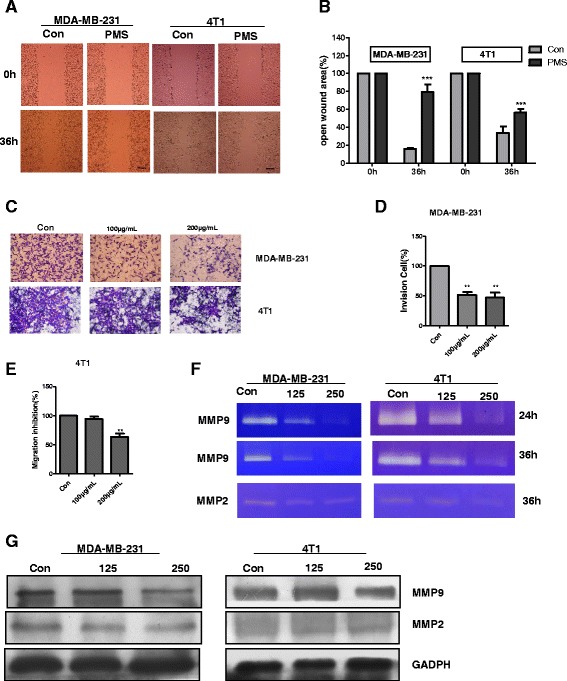
PMS inhibits migration and invasion of MDA-MB-231 or 4T1 cells by decreasing the activity of MMP insdead of the expression of MMP. **a** and **b** Effect of PMS on cellular migration by wound assay. **a** Confluent monolayers of cells were culture with solvent and with 200 μg/ mL PMS and the migration was evaluated by wound assay at 36 h. Scale bar = 100 μm. **b** The analysis of % open wound area was performed by the Tscratch software corresponding to the images in **a**. Data represent the mean ± S.D. of three independent experiments. **c–e** Effect of PMS on cellular invasion by transwell assay. **c** Cells were cultured with solvent or with 100, 200 μg/mL PMS onto the upper well coated with Matrigel. After 36 h treatment, cells passed though the Matrigel into the lower well were stained and counted. Scale bar = 25 μm (**d**) and (**e**) Analysis the % of invasion in comparison with control cell(100 %) corresponding to the images in **c**. Data represent the mean ± S.D. of three independent experiments. **f** PMS inhibits the activity of MMP9 and MMP2 secreted by MDA-MB-231 and 4T1 cells in vitro. The effect of PMS on MMP9 activity was tested by in gel zymography assay. Cells were cultured onto 6-well plates with solvent and with 125, 250 μg/mL PMS. After 24 h and/or 36 h treatment, cell supernatant was collected and performed Zymography. **g** Western Blotting showing the expression of MMP9 and MMP2 after 36 h treatment with solvent or 125, 250 μg/mL PMS. Similar results were obtained from independent experiments. The ** indicates significance different. The *** indicates extremely significance different

In the wound assay, PMS caused a decrease in the number of cells migrating into the wound area in both cell lines (Fig. 3a). The significant inhibitory effect induced by PMS at concentration of 200 μg/mL was at 36 h in comparison with control cells, where wounds were almost completely healed at this time. PMS inhibited the migration at 36 h by approximately 79.3(±8.2) %, 56.4(±4.2) % of MDA-MB-231 and 4T1 cells respectively, in comparison with control groups in which the inhibition rate were 15.8 (±1.5) %, and 33.6(±7.3) % respectively (Fig. 3b). There were significant differences between control group and PMS treatment group (*P* <0.01). However, Catechin shows no effect on the migration of tumor cells from both cell lines(Additional file 4: Figure S3A). The inhibition rate of Catehcin at 36 h were approximately 10.7(±0.9) %, 32.5(±4.2) % of MDA-MB-231 and 4T1 cells respectively, in comparison with control groups in which the inhibition rate were 15.6 (±2.0) %, and 37.1(±5.2) % respectively (Additional file 4: Figure S3B). There is no statistical significant difference between control group and Catechin treatment group.

Transwell assays showed that invasion inhibition effect of PMS on breast cancer cells were dose-dependent (Fig. 3c). At the concentration of 100 μg/mL, PMS inhibited cell migration by 48.3(±5.0) % in MDA-MB-231 cells (Fig. 3d) and 5.3(±3.5) % in 4T1 cells (Fig. 3e). Increased the concentration to 200 μg/mL the inhibition rate reached to 53 (±8.7) % in MDA-MB-231 cells (Fig. 3d) (*P* <0.01) and 36.3(±5.7) % in 4T1 cells (Fig. 3e)(*P* <0.05). However Catechin shows no inhibition ability on the invasion of cancer cells (Additional file 4: Figure S3). At the concentration of 100 μg/mL, Catechin inhibited cell migration by 0(±2.6) % in MDA-MB-231 cells (Additional file 4: Figure S3D) and 1.3(±3) % in 4T1 cells (Additional file 4: Figure S3E). Increased the concentration to 200 μg/mL the inhibition rate were 1(±3) % in MDA-MB-231 cells (Additional file 4: Figure S3D) and 1.3(±1.5) % in 4T1 cells (Additional file 4: Figure S3E).

To further detect the relationship between inhibition of PMS on cancer cell lines and the activity of MMP, the culture media of cells treated with solvent without PMS (control) or with 125 and 250 μg/mL PMS or 100 and 200 μg/mL Catechin for 24 h and/or 36 h were analyzed by gelatin zymography assay, the activity of MMP9 was dramatically decreased by PMS both in MDA-MB-231 cells and 4T1 cells shown as Fig. 3f; the activity of MMP2 was also decreased, but the decrease was less than that of MMP9 (Fig. 3f). However, Catechin has no effects on the activity of MMP9 and MMP2 shown as Additional file 4: Figure S3F. These results showed that PMS primarily inhibited the activity of MMP9 other than MMP2 to inhibit the migration and invasion of breast cancer cell lines. However, PMS didn’t change the expression of MMP9 and MMP2 protein (Fig. 3g).

### The invasion inhibition effect of PMS on MDA-MB-231 or 4T1 cells can be rescued by exogenous MMP9

**Fig. 4:**
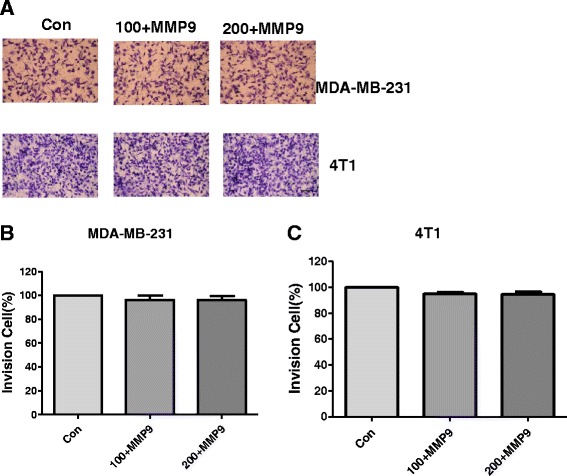
The invasion inhibition effect of PMS on MDA-MB-231 or 4T1 cells can be rescued by exogenous MMP9. **a** Cells were cultured with solvent or with 100, 200 μg/mL PMS onto the upper well coated with Matrigel. After 36 h treatment, exogenous MMP9 was added to the PMS treatment well for 36 h, cells passed though the Matrigel into the lower well were stained and counted. Scale bar = 25um. **b** and **c** Analysis the % of invasion in comparison with control cell(100 %) corresponding to the images in **a**. Data represent the mean ± S.D. of three independent experiments

In order to evaluate the relationship between the inhibition ability of PMS on MMP’s activity and tumor cells’ invasion ability, cells were cultured with solvent or with 100, 200 μg/mL PMS onto the upper well coated with Matrigel. After 36 h treatment, exogenous MMP9 was added to the PMS treatment well for 36 h, cells passed though the Matrigel into the lower well were stained and counted (Fig. 4a). After rescued by exogenous MMP9, at the concentration of 100 μg/mL, PMS inhibited cell migration by 3.7(±3.5) % in MDA-MB-231 cells (Fig. 4b) and 1.3(±3) % in 4T1 cells (Fig. 4c). Increased the concentration to 200 μg/mL the inhibition rate were 3.6(±3.2) % in MDA-MB-231 cells (Fig. 4b) and 5.3(±2.1) % in 4T1 cells (Fig. 4c).

### PMS suppressed 4T1 allograft tumor in vivo

**Fig. 5:**
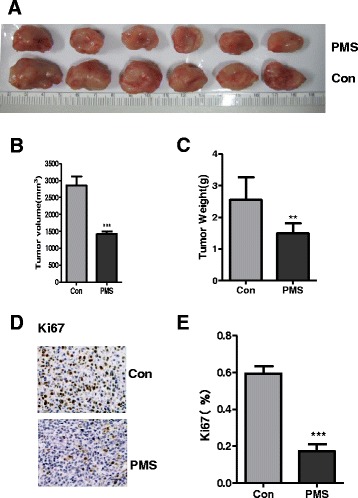
The antitumor effect of PMS treatment in vivo. Mice were injected s.c. with 1.5 × 10^6^ 4T1 cells. **a–c** The fifth day after the injection, mice were treated daily with PMS at 200 mg/kg by oral gavage for 21 consecutive days. **a** Representative tumors at the end of the experiment, (**b**) tumor volume, (**c**) tumor weight at indicated time points after treatment was calculated. **d** Paraffin-embedded sections of control or treated tumor tissues from mice were analyzed by Ki67 IHC staining. **e** Quantitative analysis of Ki67 staining corresponding to the images in **d**. Data represent the mean ± S.D. of three independent experiments. Scale bar = 25 μm. The ** indicates significance different. The *** indicates extremely significance different

As PMS inhibited cell proliferation of cancer cell lines in vitro, we expect it will further suppress tumor growth in vivo. To confirm it, 4T1 cells were injected subcutaneously into BALB/c mice to establish allograft tumors. Then the mice were treated with 200 mg/kg PMS by oral gavage for 21 consecutive days. As shown in Fig. 5a, the treatment caused significant tumor suppression. Quantitative analysis displays that the weight and volume of tumors in treatment group were less than that in control group (Fig. 5b and c) (*P* <0.05). Immunohistochemistry staining of ki67 further conformed that tumor growth was synergistically inhibited (Fig. 5d), and the difference between these two groups was significant (Fig. 5e).

### The antitumor effect of PMS was achieved via decreasing angiogenesis

4T1 cell line is a malignant rodent breast cancer cell line which can cause severe lung metastasis. Since PMS inhibited the growth of the primary tumor by significantly reducing the volume and weight of tumors, in order to further explore the effect of PMS on inhibiting metastasis, we compared several indicators of pulmonary metastasis between the control group and PMS treatment group.

**Fig. 6:**
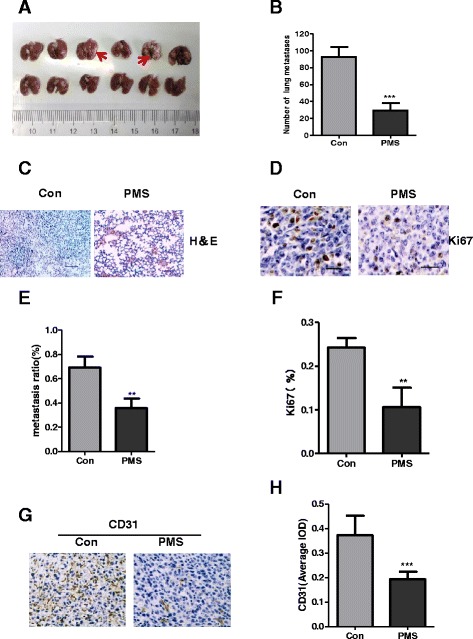
PMS inhibited tumor metastasis via decreasing angiogenesis. The complete lungs were fixed after autopsy and the number of lung metastases foci (*arrow*) was counted (**a**). **b** Quantitative analysis of metastases corresponding to the images in **a**. Paraffin-embedded sections of control or treated lung tissues from mice were analyzed by (**c**) H&E staining (Scale bar = 100 μm) and analyzed by (**d**) Ki67 IHC staining(Scale bar = 25 μm). **e** and **f** Quantitative analysis of metastases or Ki67 staining corresponding to the images in **a** and **b** respectively. Data represent the mean ± S.D. of three independent experiments. **g** Paraffin-embedded sections of control or treated tumor tissues from mice were analyzed by CD31 IHC staining. Scale bar = 25 μm. **h** Quantitative analysis of CD31 staining corresponding to the images in **g**. Data represent the mean ± S.D. of three independent experiments. The ** indicates significance different. The *** indicates extremely significance different

The complete lungs were fixed after autopsy and the number of lung metastases was counted, Fig. 6a shows that the metastasis foci (arrow) of the control group(92.8 ± 11.9) were significantly more than those of the PMS treatment group(29.5 ± 8.4), and there were statistically significant differences between the two groups (Fig. 6b) (*P* <0.01). Lung tissue were stained by H&E, the lung metastasis in the control group(69.3 % ± 9.0 %) were much bigger than those in PMS treatment group (36.0 % ± 7.5 %) (Fig. 6c), quantitative statistics existed significant differences (Fig. 6e) (*P* <0.05). The IHC staining of Ki67 of control group (24.3 % ± 2.1 %) is significantly more than PMS treatment group (10.6 % ± 4.5 %) (Fig. 6d and f) (*P* <0.05).

To further explore the mechanism of PMS inhibition on tumor lung metastasis, we detected the biomarker of angiogenesis-CD31 in primary tumor tissues by IHC staining (Fig. 6g), after IPP software semi quantitative analysis, there were significant differences between the two groups (Fig. 6h) (*P* <0.01)(control vs. PMS is 0.37 ± 0.08 vs.0.19 ± 0.03). It can be inferred that PMS may inhibits activity of MMP9 and MMP2 which causes angiogenesis decreasing, therefore reduces lung metastasis.

## Discussions

As potential cancer therapeutic and preventive agents, herbal medicine is currently becoming more and more attentive. In a cross-sectional survey, a substantial number of people with cancer are likely to be taking herbal medicine [20]. Previous studies proved that anti-cancer effect of herbal medicine in various tumors: Chinese herbal medicine Qingyihuaji Formula (QYHJ) could inhibit pancreatic cancer cell invasion and metastasis in part by reversing tumor-supporting inflammation [21]; Ginseng can be used as an anti-cancer agent in the treatment of colorectal Cancer [22]; Withaferin A (WFA) induces breast cancer growth inhibition [23]. Now more than 60 herbal complexes are being studied as anti-cancer medicine. Plant derived anticancer agents in clinical use can be divided into four important groups: Vinca, Alkaloids, Taxanes, podophyllotoxin [24], so it is feasible to find new drugs that inhibit the metastasis of breast cancer from the traditional Chinese herbal medicine. Depending on this standpoint, we firstly screened PMS targeted binding to MMP9 via docking calculation (Fig. 1b). PMS, a major effective elements extracted from Plantago major L, is applied to the treatment of many diseases, such as its protective activities against Cd-induced renal injury [15], anti-inflammation effect [25], anti diabetic effect [26]. Plantago major L has an inhibitory effect on some tumor (In vivo Antitumoral Effect of Plantago major L. Extract on BALB/C Mouse with Ehrlich Ascites Tumor), however which component play the main role is not clear. We expected that the PMS may have potential anti-tumor effects.

To demonstrate this prediction, first of all, we evaluated the effect of PMS on cancer cell lines. Our results show that PMS can inhibit the growth of cancer cells (Fig. 2) without side effect on normal cells (Additional file 3: Figure S2d), as well as their ability of invasion and metastasis, while reducing the MMP9 and MMP2 activity (Fig. 3). As a negative control, Catechin can also inhibit the growth of cancer cells (Additional file 3: Figure S2A-C), but has no inhibition effects on the migration and invasion of cancer cells, either the activity of MMP2 and MMP9 (Additional file 4: Figure S3). It indicates that the dose we choose to treat cells in migration and invasion assays would not decrease the cell viability. Therefore, PMS reducing the migration and invasion ability of cancer cells is not due to the decreasing of cell viability. Exogenous MMP9 can rescue the invasion ability of cancer cells that treated with PMS (Fig. 4). So PMS did inhibit tumor cell proliferation and migration and invasion activity by decreasing the activity of MMP.

Due to the wide range of MMPs activities in tumor, there is not an exact conclusion on the mechanism of MMP promoting metastasis. Some studies suggest that it’s related to ECM degradation. Increased MMP activity results in both matrix remodeling and release of chemokines, cytokines and growth factors trapped within the ECM [27], promoting EMT process-KLF8-to-MMP9 signaling that promotes human breast cancer invasion [28]. Others argue that it’s involved in regulating other receptor protein expression-SPARC and MMP9 interact to regulate tumor metastasis [29].and controlling vascular formation. Tumor cell-produced MMP9 promotes vessel formation in an orthotopic allograft model of basal-like triple negative breast cancer [9]. Obviously, further study on the concrete mechanism of PMS inhibiting tumor via decreasing MMP activity are warrant.

In order to further determine the inhibition of PMS on tumor in vivo, we carried out tumor inhibition experiments in vivo. 4T1 cells were chosen to allograft, because the 4T1 tumor is highly tumorigenic and invasive, unlike most tumor models, can spontaneously metastasize from the primary tumor in the mammary gland to multiple distant sites including lymph nodes, blood, liver, lung, brain, and bone [30, 31], The progressive spread of 4T1 metastases is very similar to that of human breast cancer. Our study confirmed that only 1.5 × 10^6^ cells can bear tumor successfully (Fig. 5a), and led lung metastasis (Fig. 6a), similar to other researcher’s previous studies [32].

Tumor bearing mice were treated by oral administration of PMS. There are not many studies on the PMS dose of oral administration in mice. In the most researches, the whole herb was give, such as Plantago asiatica decoction. Rats were orally administered with the dose of 10 g/kg of Plantago asiatica in an Pharmacokinetics of plantamajoside from Plantago asiatica [18]. Based on the total body surface area of the mouse, using 0.14 as the conversion factor [33], mice should be given 14 g/kg Plantago asiatica. And the content of PMS in the whole herb was about 0.03 [34], so the dose of PMS given mouse should be 420 mg/kg. What’s more, other report said no chronic toxicity was observed for plantamajoside concentrate even at a dose of 2000 mg/kg intragastrically in rats [35], this dose may reach 2800 mg/kg in mouse. However, the higher dose of PMS was 40 mg/kg(56 mg/kg in mouse) in an study on nephroprotection of PMS in rats [19]. So we compromised choose the dose 200 mg/kg, and get significant results. After necropsy, we found that PMS inhibits the lungs metastasis of 4T1 tumor cells (Fig. 6). According to the starting point of the current research—inhibiting MMP activity, and studies have shown that MMP control the angiogenic switch [36]; as it’s widely known that angiogenesis and tumor metastasis are closely related [37]. In breast cancer, many secretory or cell surface proteins implicated in cell homing to bone, angiogenesis, invasion, and osteoclast recruitment influence the tumor microenvironment in favor of metastasis [38], so we explore the mechanism of PMS inhibition by detecting angiogenesis. CD31 can be used as a marker of density of microvessel [39]. As Fig. 6 shows, the expression of CD31 by IHC staining has significant differences between control groups and PMS treatment group. PMS significantly decreased vascular density in 4T1 tumor.

We first demonstrated the antitumor effect of PMS, and it is likely to be achieved by decreasing angiogenesis which may be regulated by MMP9 and MMP2 activity. Further identification should be done to determine the exact mechanism of the inhibition of PMS. Generally, Chinese herbal medicine is applied as a component of adjuvant therapy for cancer. In our study, PMS was used as a single anti-tumor agent and expressed anticancer effect. In sum, PMS is a promising therapeutic agent for breast cancer, though its effectiveness on tumor inhibition combined with other chemotherapeutic agents should be explored.

## Conclusions

Our results not only pointed out that PMS significantly restricted allograft tumor growth at the concentrations chosen, but also demonstrate that the inhibition effect of PMS on MMP9/MMP2 activity may contribute to its anti-tumor effects.

## Additional files

Additional file 1: Table S1. The molecular docking of different agents with MMP9. Molecular docking of Catechin, Chrysophanol,Phlorizin, Salidroside, Curcumin, Isoacteoside, Silymarin, Echinacoside, Gastrodin, Harpagoside with MMP9. All of ∆G are higher than -10.38 cal/mol(the ∆G of PMS with MMP9). (PPTX 50 kb) Additional file 2: Figure S1. Different agents affect the activity of MMP9. Detected IOD of substrate activated by MMP9 treated with solvent or these ten agents. Data represent the mean ± S.D. of three independent experiments. (PPTX 106 kb) Additional file 3: Figure S2. Cell viability decreased after treatment of Catechin and PMS cannot change the cell viability of CHO cell line. Cell viability was analyzed using Cell Counting Kit 8 at 0, 12, 24, 36, 48 h after 25, 50, 100, 200, 400 μg/m L Catechin treatment in (A) MDA-MB-231 and (B) 4T1 cells. Data represent the mean ± S.D. of three independent experiments. (C) Colony formation of MDA-MB-231 and 4T1 cells. Cells were treated with 100 μg/mL Catechin for 36 h, followed with crystal violet staining of attached cells at 10 days. Similar results were obtained from independent experiments. D PMS has no side effect on CHO. There was no significant change of cell viability between pre and post 300 μg/ml PMS treatment for 36 h. (PPTX 982 kb) Additional file 4: Figure S3 Catechin shows no inhibition effects on migration and invasion of MDA-MB-231 or 4T1 cells and no inhibition the activity of MMP2 and MMP9. Effect of Catechin on cellular migration by wound assay.(A) Confluent monolayers of cells were culture with solvent and with 200 μg/ mL Catechin and the migration was evaluated by wound assay at 36 h. Scale bar = 100 um. (B) The analysis of % open wound area was performed by the Tscratch software corresponding to the images in A. Data represent the mean ± S.D. of three independent experiments. C–E. Effect of Catechin on cellular invasion by transwell assay. (C) Cells were cultured with solvent or with 100, 200 μg/mL catechin onto the upper well coated with Matrigel. After 36 h treatment, cells passed though the Matrigel into the lower well were stained and counted. Scale bar = 25um (D) and (E) Analysis the % of invasion in comparison with control cell(100 %) corresponding to the images in C. Data represent the mean ± S.D. of three independent experiments. F. The activity of MMP9 and MMP2 secreted by MDA-MB-231 and 4T1 cells kept same after Catechin treating in vitro. The effect of Catechin on MMP activity was tested by in gel zymography assay. Cells were cultured onto 6-well plates with solvent and. After 36 h treatment, cell supernatant was collected and performed Zymography. Similar results were obtained from independent experiments. (PPTX 1293 kb)
