# Supplementary figures and images for: Plantamajoside, a potential anti-tumor herbal medicine inhibits breast cancer growth and pulmonary metastasis by decreasing the activity of matrix metalloproteinase-9 and -2
Source: BMC Cancer. 2015 Dec 16;15:965. doi: 10.1186/s12885-015-1960-z (PMC4682252; doi:10.1186/s12885-015-1960-z)

## Slide 1
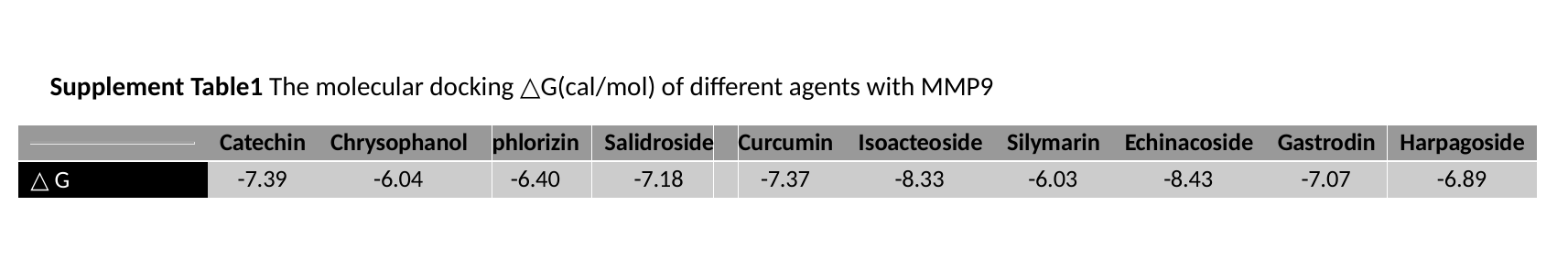

Supplement Table1 The molecular docking △G(cal/mol) of different agents with MMP9

Supplement: Additional file 1: Table S1. — The molecular docking of different agents with MMP9. Molecular docking of Catechin, Chrysophanol,Phlorizin, Salidroside, Curcumin, Isoacteoside, Silymarin, Echinacoside, Gastrodin, Harpagoside with MMP9. All of ∆G are higher than -10.38 cal/mol(the ∆G of PMS with MMP9). (PPTX 50 kb) [file 12885_2015_1960_MOESM1_ESM.pptx]

## Slide 1
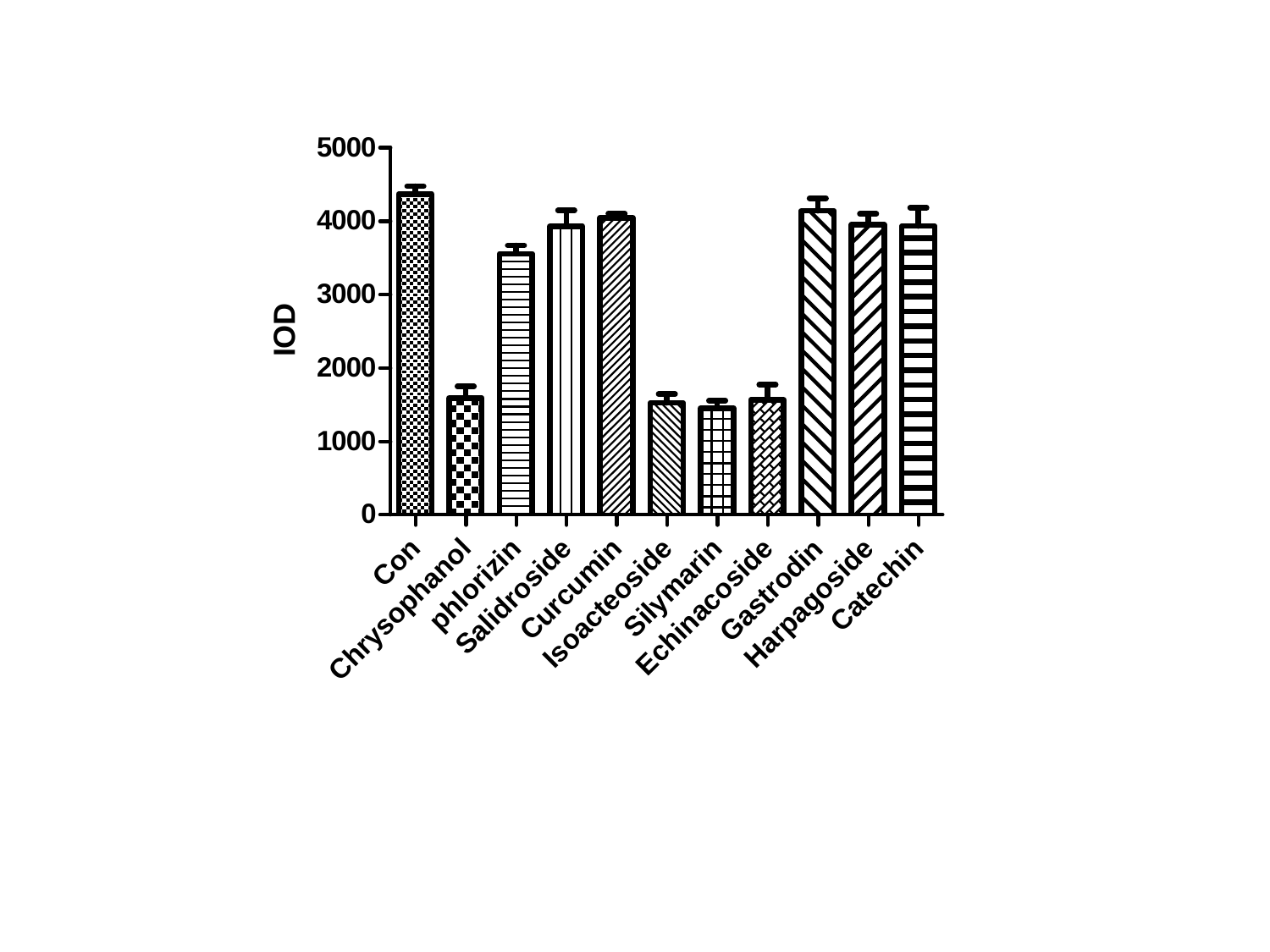

Supplement: Additional file 2: Figure S1. — Different agents affect the activity of MMP9. Detected IOD of substrate activated by MMP9 treated with solvent or these ten agents. Data represent the mean ± S.D. of three independent experiments. (PPTX 106 kb) [file 12885_2015_1960_MOESM2_ESM.pptx]

## Slide 1
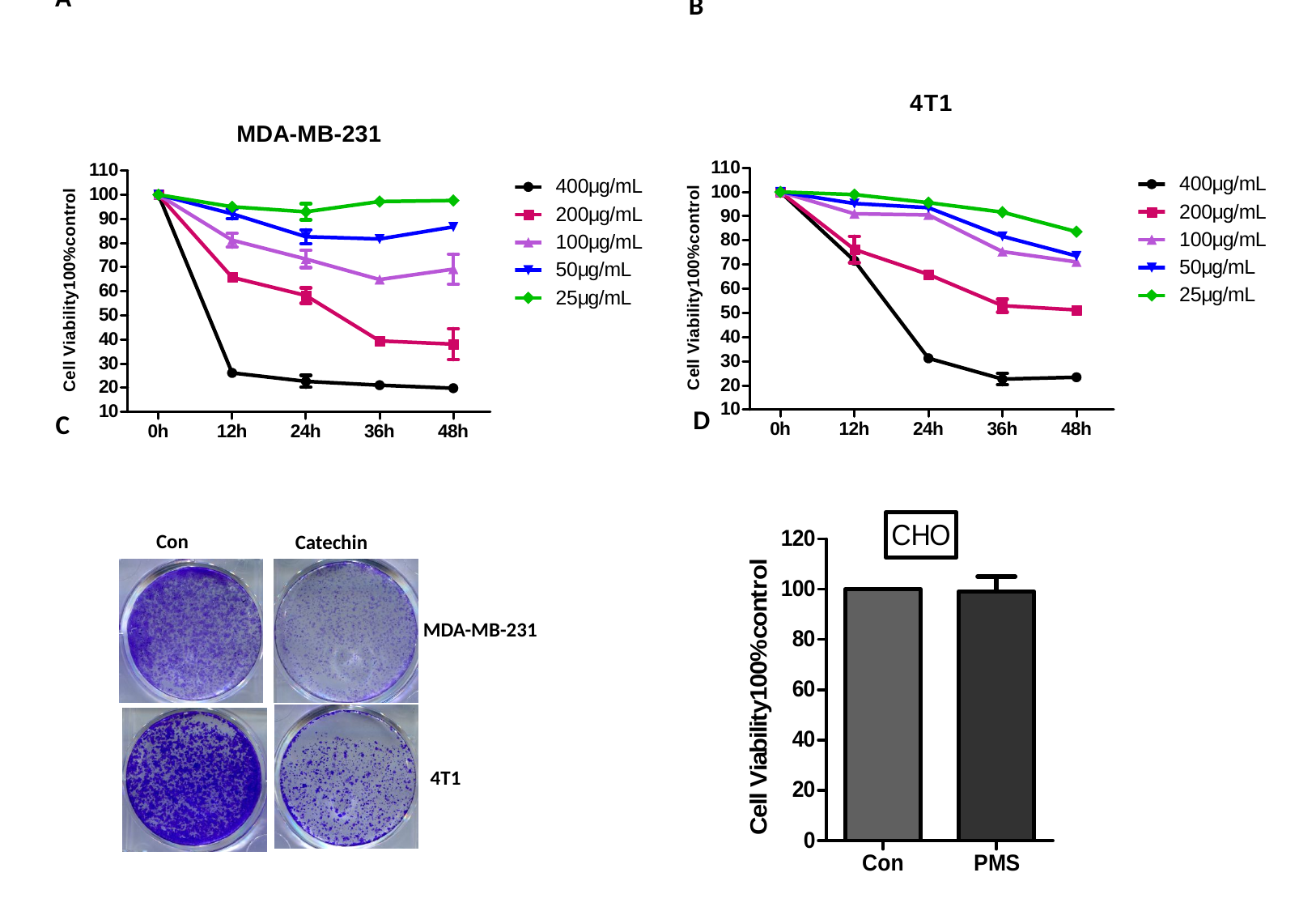

A
B
D
C
Con
Catechin
MDA-MB-231
4T1

Supplement: Additional file 3: Figure S2. — Cell viability decreased after treatment of Catechin and PMS cannot change the cell viability of CHO cell line. Cell viability was analyzed using Cell Counting Kit 8 at 0, 12, 24, 36, 48 h after 25, 50, 100, 200, 400 μg/m L Catechin treatment in (A) MDA-MB-231 and (B) 4T1 cells. Data represent the mean ± S.D. of three independent experiments. (C) Colony formation of MDA-MB-231 and 4T1 cells. Cells were treated with 100 μg/mL Catechin for 36 h, followed with crystal violet staining of attached cells at 10 days. Similar results were obtained from independent experiments. D PMS has no side effect on CHO. There was no significant change of cell viability between pre and post 300 μg/ml PMS treatment for 36 h. (PPTX 982 kb) [file 12885_2015_1960_MOESM3_ESM.pptx]

## Slide 1
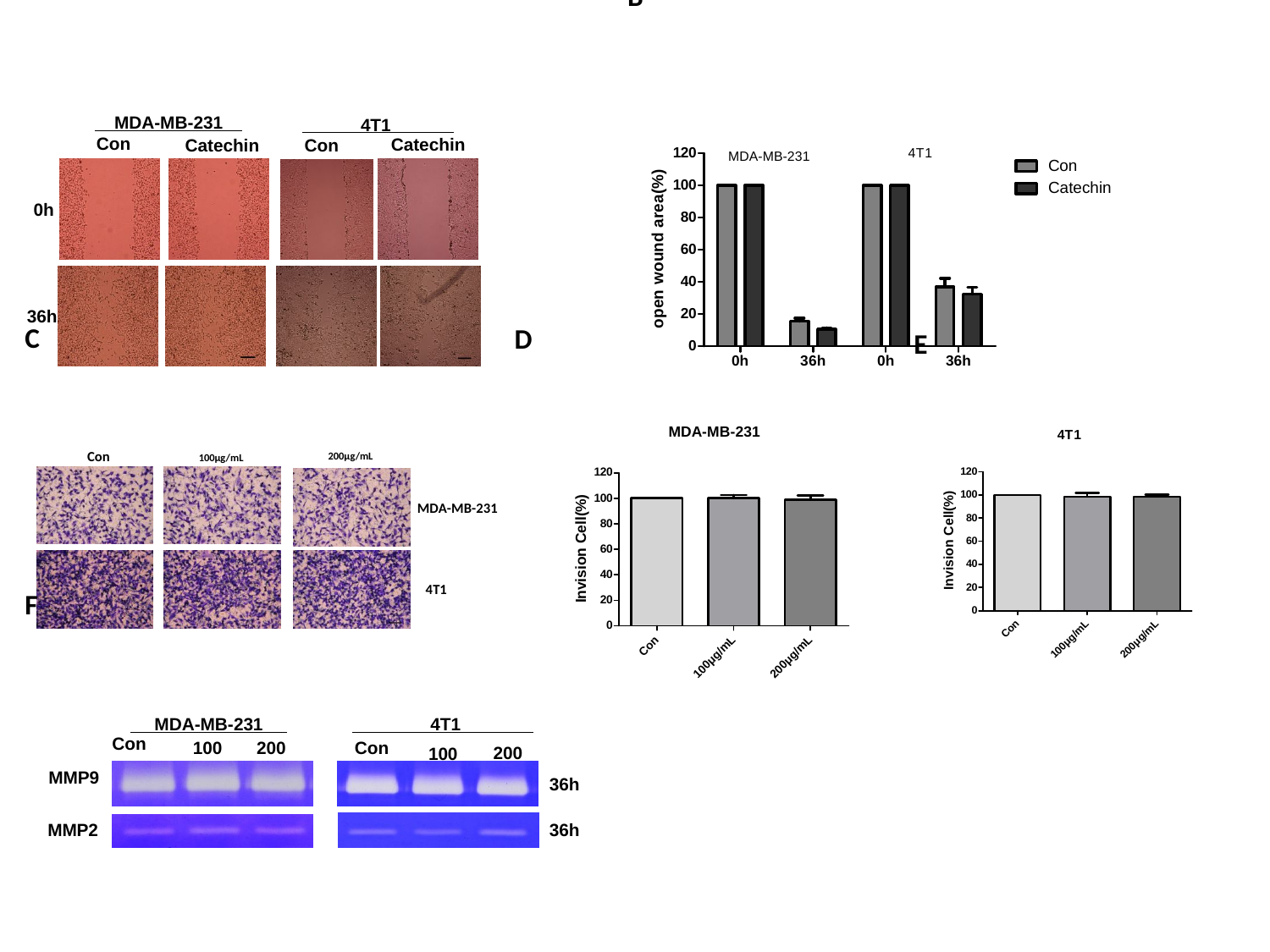

A
 MDA-MB-231
 4T1
Con
Catechin
Con
Catechin
0h
36h
B
 C
 D
 E
Con
200μg/mL
100μg/mL
MDA-MB-231
4T1
 F
 4T1
 MDA-MB-231
Con
Con
100
200
200
100
MMP9
36h
MMP2
36h

Supplement: Additional file 4: Figure S3 — Catechin shows no inhibition effects on migration and invasion of MDA-MB-231 or 4T1 cells and no inhibition the activity of MMP2 and MMP9. Effect of Catechin on cellular migration by wound assay.(A) Confluent monolayers of cells were culture with solvent and with 200 μg/ mL Catechin and the migration was evaluated by wound assay at 36 h. Scale bar = 100 um. (B) The analysis of % open wound area was performed by the Tscratch software corresponding to the images in A. Data represent the mean ± S.D. of three independent experiments. C–E. Effect of Catechin on cellular invasion by transwell assay. (C) Cells were cultured with solvent or with 100, 200 μg/mL catechin onto the upper well coated with Matrigel. After 36 h treatment, cells passed though the Matrigel into the lower well were stained and counted. Scale bar = 25um (D) and (E) Analysis the % of invasion in comparison with control cell(100 %) corresponding to the images in C. Data represent the mean ± S.D. of three independent experiments. F. The activity of MMP9 and MMP2 secreted by MDA-MB-231 and 4T1 cells kept same after Catechin treating in vitro. The effect of Catechin on MMP activity was tested by in gel zymography assay. Cells were cultured onto 6-well plates with solvent and. After 36 h treatment, cell supernatant was collected and performed Zymography. Similar results were obtained from independent experiments. (PPTX 1293 kb) [file 12885_2015_1960_MOESM4_ESM.pptx]
